# Novel Phlebovirus in Febrile Child, Greece

**DOI:** 10.3201/eid1705.101958

**Published:** 2011-05

**Authors:** Vassiliki Anagnostou, Grigorios Pardalos, Miranda Athanasiou-Metaxa, Anna Papa

**Affiliations:** Author affiliations: Aristotle University of Thessaloniki Thessaloniki, Greece (V. Anagnostou, A. Papa);; Hippokration Hospital, Thessaloniki (G. Pardalos, M. Athanasiou-Metaxa)

**Keywords:** Phlebovirus, Adria virus, sandfly fever, Greece, viruses, vector-borne infections, letter

**To the Editor:** Phleboviruses (family *Bunyaviridae*, genus *Phlebovirus*) are arthropod-borne, single-stranded, RNA viruses. Their genome consists of 3 segments—small, medium, and large—which encode the nucleoprotein and nonstructural proteins, the envelope glycoproteins, and the viral polymerase, respectively (1). The genus *Phlebovirus* consists at least 60 antigenically distinct serotypes, including the sandfly fever viruses transmitted to humans by phlebotomine sandflies. In the Mediterranean region, 3 phleboviruses are known to circulate: Toscana virus, sandfly fever Naples virus, and sandfly fever Sicilian virus. Sandfly fever Naples virus and sandfly fever Sicilian virus cause a transient febrile illness, whereas Toscana virus is sometimes neurovirulent, leading to aseptic meningitis and meningoencephalitis (2,3).

Phleboviruses have been detected in Greece in clinically ill persons and in sandflies; seroprevalance in humans is high, especially in the Ionian islands (3–7). In addition, in 2002, a Sicilian-like virus (Cyprus virus) was responsible for a major outbreak of febrile illness among Greek Army forces in Cyprus (8). We report genetic detection and sequencing of an Adria virus from a boy who was hospitalized because of simple febrile seizure; the identical sequence was initially detected in sandflies collected in a coastal area in Albania.

On September 23, 2009, a 2.5-year-old boy was admitted to the Hippokration Hospital of Thessaloniki, Greece, after a single episode of simple febrile seizure. The patient was febrile (38.2°C) and had vomited 1 time while in nursery school. He had sudden onset of eye gaze, perioral cyanosis, masseter muscle spasm, generalized tonic convulsions of the body and extremities, and involuntary loss of urine. The episode lasted ≈3 minutes, after which the child became irritable and sleepy.

At the time of hospital admission (30 minutes later), he was afebrile and conscious. The boy’s history contained no previous neurologic or developmental disabilities and no family history of epilepsy or febrile seizures. Clinical examination, which included a thorough general and detailed neurologic evaluation, revealed no abnormalities except mild rhinitis. Laboratory tests showed leukocytosis (22,600 cells/μL) with 85.7% neutrophils. Blood levels of electrolytes, urea nitrogen, creatinine, glucose, albumin, bilirubin, alkaline phosphatase, and aminotransferases; prothrombin time; and urinalysis results were within reference limits. Electroencephalogram showed no brain abnormalities. Lumbar puncture and neuroimaging were not considered necessary.

After 2 days of hospitalization, the child recovered, was free of signs and symptoms, and was discharged from the hospital with a diagnosis of simple febrile seizure and mild upper respiratory infection. According to a report from his mother 1 year later, the child remains well without any recurrence of febrile or other type of seizures.

Viral RNA was extracted from the patient’s blood sample taken at the time of hospital admission. Nested reverse transcription–PCR using degenerate primers was applied to amplify a 222-bp fragment of the large RNA segment of phleboviruses (9). The retrieved sequence was identical to sequences detected in sandflies collected in 2005 in the Adriatic coastal region of Albania; that strain was provisionally named Adria virus (10). Adria virus is distinct from other recognized members of the genus *Phlebovirus* and clusters with phleboviruses of the Salehabad serocomplex, such as Salehabad virus and Arbia virus, differing by 21.6% and 29.6% with Salehabad virus and Arbia virus at the nucleotide level and by 3% and 17.7% at the amino acid level, respectively (Table).

**Table:** Percentage nucleotide and amino acid sequence divergence among phleboviruses*

Virus	Virus (GenBank accession no.)						
	ADRV- GR	ADRV (HM043726)	SALV (GU143716)	ARBV (DQ862467)	SFSV (EF095551)	TOSV (FJ153280)	SFNV (EF095548)
ADRV-GR		0	21.6	29.6	82.0	84.8	96.4
ADRV	0		21.6	29.6	82.0	84.8	96.4
SALV	3.0	3.0		33.5	83.6	89.8	100
ARBV	17.7	17.7	17.5		75.5	73.6	96.4
SFSV	85.6	85.6	85.6	82.3		76.1	70.7
TOSV	78.3	78.3	76.3	73.1	75.7		47.4
SFNV	86.7	86.7	86.2	87.0	84.1	29.2	

*Values above the diagonal are nucleotide sequence divergence, and values below the diagonal are amino acid sequence divergence, estimated by neighbor-joining method. ADRV-GR, Adria virus from febrile child in Greece, 2009; ADRV, Adria virus; SALV, Salehabad virus; ARBV, Arbia virus; SFSV, sandfly fever Sicilian virus; TOSV, Toscana virus; SFNV, sandfly fever Naples virus.

Detection of the Adria virus sequence in the patient’s blood suggests that this virus is pathogenic to humans. As expected, serologic testing of the sample taken at the time of admission produced negative results for phleboviruses; a convalescent-phase blood sample was not available. Although the course of the disease in the child was mild, further studies will show the role of this strain in public health.

Because the duration of viremia in persons with phlebovirus infections is short, use of molecular methods for the laboratory diagnosis of phleboviral infections is limited; and even when a phleboviral infection is confirmed by serologic testing, the exact strain is difficult to determine. Physicians in Greece, as in other Mediterranean countries, should be aware of the circulation of phleboviruses and potential risk for phlebovirus-associated infections during the summer. Such infections, especially with neurologic signs, should be included in the differential diagnosis of summer febrile syndromes.
